# A Plea for Uterine Curettage as a Routine Measure after Abortion before the Third Month1Read before the section on gynecology and obstetrics of the Buffalo Academy of Medicine, April 27, 1897.

**Published:** 1897-08

**Authors:** Sigmund Goldberg

**Affiliations:** Buffalo, N. Y.


### A PLEA FOR UTERINE CURETTAGE AS A ROUTINE MEASURE AFTER ABORTION BEFORE THE THIRD MONTH.[1]

By SIGMUND GOLDBERG, M. D., Buffalo, N. Y.

THE frequency with which abortion is followed by death, or a life of physical ailments, more or less severe, is my apology for the presentation of this paper. If I shall be successful in arousing a discussion in the line of prophylaxis as here outlined I shall feel that it has not been in vain. Though fatal results after abortion are rare, and when occurring due to sepsis rather than to hemorrhage, yet the fact remains that deaths do occur and that

1. Read before the section on gynecology and obstetrics of the Buffalo Academy of Medicine, April 27, 1897.

very fact merits the most profound consideration of any method which may have a tendency to lessen the number. Much of female suffering is directly traceable to this accident. The pathological interruption of pregnancy, as uterine and other pelvic diseases, especially subinvolution and consequent displacement, diseases of the endometrium and connective tissue result from abortion sterility as well. These diseases leave their traces indelibly marked upon the system of woman.

The accident of abortion, when fully inaugurated, can but seldom be interfered with. The question naturally arises, How shall it be treated to the best advantage and its sequelæ often so dire be avoided? The bad results are in the main due to the retention of parts of ovum or membranes, and I believe it impossible for the attendant always to be absolutely certain that the uterus is completely emptied. We cannot be so sure of a thoroughly retracted uterus after abortion in the early months as after labor at term. The uterus in its physiological post partem condition is better able to expel retained products of conception, be it ever so small, than a uterus in the pathological condition during or following abortion. The indications of a condition of the uterus which will almost surely lead to a long list of physical ailments on the part of the patient, will be hemorrhage or sepsis, or both. Now, is it right and always safe to wait for these symptoms before resorting to the only treatment which is admissible to its proper management? It has come to my knowledge only too often that physicians temporise with drugs, whose only effect is to contract the os uteri and thus defeat the very object it was intended to accomplish. It is my aim to advocate uterine curettage after every case as a routine measure without waiting for indications which make the operation imperative. Ever since the curette was first introduced by Recamier, in 1846, it has become exceedingly popular, both in its original and various modified forms. Many authorities pronounced the practice barbarous and unscientific—Chassaignac, Becquerel, Dubois, Scanzoni, Credé—but in vain, the curette maintained its fame and increased in popularity. But its greatest fame, in my humble opinion, will be attained only when it is generally used for the prevention of those very conditions which are now held to be the only indications for resort thereto. During the past four years it has been my custom to curette every uterus after abortion, no matter what the period of utero-gestation or the condition of the patient. I have never had cause to regret it, but, on the contrary, have felt that my patient was safe. The operation is perfectly safe, and any physician competent to manage abortion should be able to do this minor procedure. I always use the Sims sharp curette. Many accidents have been attributed to it. Recamier himself met with three cases of death from perforation of the uterus by his curette; Dumarquay with two. Chamberlain, of New York, saw a case of hysterical tetanus therefrom; Peaslee, a death from collapse; Thomas, a narrow escape from the same cause; Parker, a case of peritonitis. But in these cases it was not the Sims sharp curette which was used, but the dangerous Recamier's curette. The Sims sharp curette should, of course, be handled with ordinary common sense in order not to cut too deeply and perhaps perforate the uterine wall, as happened to Spiegleberg and to Chroback while curetting a sarcoma of the uterine cavity. But this is not the condition of which we speak.

Mundé says that only unjustifiable brutal force could possibly perforate a normal uterine wall with a Sims curette. If, then, we understand curettage of the uterine cavity after abortion in every case to be a prophylactic measure against hemorrhage and sepsis, if properly performed, that is safe, there can be no just reason for waiting for the development of symptoms which may be the first indication that we have not done our full duty to our patients. The operation is easily performed, as after abortion there is always sufficient dilatation for its accomplishment. I almost always do it without an anesthetic and frequently without saying anything to the patient about it. The curette is the only instrument that will thoroughly clean out the cavity of the uterus. Many operators advocate the use of the finger for clearing the uterus, but I hold the hand is cramped and cannot work to the same advantage; besides, it is much more painful to the patient and I can endorse fully from my own experience what is said on this point by Dr. Alfred C. Carpenter, *Medical Record*, May 2, 1896, who, after referring to the use of the finger, says it is claimed the curette is dangerous, liable to puncture the fundus, that it cuts away too much uterine tissue and opens the uterine sinuses, allowing sources for infection. To be sure, a great deal of injury can be done with the curette. Indeed, I would never advocate the introduction of any instrument into such a uterus by an unskilled hand, but in the hand of one who knows how to use it, it can be used with just as much safety and to better advantage. Now, as a rule, I do inform the patient and family that it is necessary to clean out the uterus. If there exists absolute confidence in the judgment and skill of the attendant, it will not be difficult to overcome any feeling of fear or prejudice that the patient or friends may entertain. Complications of a more or less serious nature are bound to follow if the uterus is not properly cleaned, which the patient will very readily believe to be the direct result of some little detail or other in the treatment.

The one thing indicated and the measure that will avoid all trouble is to clean out the organ and remove all elements of danger. If you state positively what line of action is required, and the family is not guided by your advice you remain true to your principles, guard against inuendoes and maintain an impregnable position if you withdraw from the case or place the responsibility on the family. A physician has no more right to wait for a rise of temperature or the development of a fungoid endometritis before removing decidual or placental tissue, than he has to wait for evidences of sepsis or sloughing before cleaning a lacerated or contused wound of foreign matter. It is clearly his duty to insure a surgically clean surface. In a brochure recently issued by Conley, of New York, he says the many cases of subinvolution, metritis, tubal trouble and inflammation about the uterus, not to speak of the violent acute attacks that have come under the writer's notice, has often led him to wonder if the average physician really appreciated the importance of thoroughly removing the products of conception. How many of the women who are today suffering from pelvic disease, and who date their trouble at the time of a premature birth, owe their condition to the ignorance or indifference of their attendant ? To the one who is in a position to know that with exercise of due care and skill the woman who is in a reasonably good condition can pass through an accident of this kind with but a nominal risk, what comfort is brought. And what satisfactory results are possible in all cases when the proper technique is followed out. This is a picture that is indeed a painful reproach to the skill and conscience of the profession.

That sepsis and not cold, or a too early getting up, or any of the many other reasons evolved in the mind of the attendant or the patient herself, is the direct cause of the immediate or remote ill results in most cases, is clear to the mind of every intelligent physician. I have records of forty-seven cases curetted during the four years past. One of this number I have curetted six times and others more than once. It seems to be the special privilege of the woman living in that section of our city in which my lot is cast to treasure almost as tenderly as a fond mother does the most delicate of her offspring, a male catheter, which she brings into requisition after each period missed. This is a sufficient number in the practice of one man to testify to the uniform efficiency of the management advocated. How much more agreeable, as Conley says, is this state of things than any one of the many gruesome pictures that is liable to meet the physician's gaze when called to a patient who has been subjected to the so-called conservative plan of treatment. An alarming hemorrhage, due to a fungoid endometritis, subinvolution, with all its vague symptoms, misplacement, with its attending discomforts, purulent endometritis, cellulitis and pelvic abscess, tubal and ovarian inflammation, violent septic peritonitis, profound septic absorption, and metastatic deposits, are results that serve only to condemn any other mode of procedure than the one that is clearly indicated. An ounce of prevention, etc., appeals fully as strongly to the physician in attendance on a case of abortion as in any other professional relationship.

508 Eagle Street.

